# Supplementary material for: Interleukin-6 and Lymphocyte-to-Monocyte Ratio Indices Identify Patients with Intrahepatic Cholangiocarcinoma
Source: Biomedicines. 2024 Apr 11;12(4):844. doi: 10.3390/biomedicines12040844 (PMC11047984; doi:10.3390/biomedicines12040844)
Supplement: Supplementary file 1 [file biomedicines-12-00844-s001.zip › biomedicines-2911121-supplementary.pdf]

Supplementary material

Isolation of peripheral mononuclear cells (PBMCs)

In brief, blood samples were diluted with 1x phosphate buffer saline (PBS, pH 7.4) and separated into PBMCs using Ficoll-Paque™ (GE Healthcare, NJ, USA). The mononuclear cell layer (lymphocytes, monocytes, and thrombocytes) was obtained at the interphase through centrifugation (400xg, 30 min, 20 °C). The mononuclear cell layer was transferred to a new conical tube and 1xPBS was added. The mixed mononuclear cell layer was centrifuged (300g, 10 min, 20 °C) and the cells from the granules were suspended in 1xPBS. After centrifugation (200xg, 10 min, 20 °C), the supernatant was extracted. The number of cells was determined using a cell counter. A $5x10^6$ cells/ml suspension was prepared in a complete medium and incubated at 37 °C for three hours.

Extraction of RNA

RNA was extracted from PBMCs using TRIzol™ (Thermo-Fisher Scientific, MA, USA). Fifty microlitre of chloroform was applied to PBMCs before homogenization (Scilogex D500, CT, USA). The homogenate was combined and centrifuged (12,000xg, 15 min) and the supernatant was transferred to a new tube. Isopropanol (200 µL) was added, mixed, and centrifuged (12,000xg, 15 min, 4 °C) to separate supernatant. The RNA pellets were treated with 1 mL of 100% ethanol, then dried. The RNA pellets were dissolved in 20 µL of DEPC water, and the total amount of RNA was measured using a spectrophotometer (Thermo-Fisher Scientific, MA, United States) set to 260 nm.

Synthesis of cDNA

cDNA was synthesized using the Super-Script® III First-Strand Synthesis System for RT-PCR. In brief, 2 µL of RNA from PBMCs (500 ng/µL), 0.5 µl of 50 µM oligo (dT), and 10 µM of dNTP mix were mixed and incubated at 65 °C for 5 min. The mixture was placed on ice promptly for 1 min. Two microlitre of 10x RT buffer, 4 µL of 25 mM $MgCl_2$, 2 µL of 0.1 M DTT, 1 µL of RNase OUT (40 U/µL), and 1 µL of Super-Script® III RT (200 U/µl) were introduced to the cDNA synthesis mixture. Each RNA template was mixed with 10 µL of the cDNA synthesis mixture before a 50-min incubation (50 °C) and reaction termination (incubation at 85 °C for 5 min). Each tube was incubated with 1 µL of RNaseH at 37 °C for 20 min. The synthesized cDNA was kept at 20 °C until use.

Quantification of mRNA expression of IL-6 and TNF-α

Serum concentrations of IL-6 and TNF-α were determined using iTaq™ Universal SYBR™ Green Super-mix (Bio-Rad Laboratories Inc., California, United States). The IL-6 and TNF-α mRNA expression levels were normalized using glyceraldehyde-3-phosphate dehydrogenase (GAPDH). cDNA was

amplified on a Bio-Rad CFX (Bio-Rad, CA, USA) using the forward and reverse primer sequences for IL-6 and TNF-α. The real-time PCR condition consisted of 46 cycles of denaturation (95°C, 10 sec), annealing (60°C, 10 sec), and extension (72°C, 20 sec). Each analysis was done in triplicate across three independent experiments.

Gene name	Forward primer	Reverse primer
IL6	GTACATCCTCGACGGCATC	AGCCACTGGTTCTGTGCCT
TNF-α	TGCTTGTTCCTCAGCCTCTT	ATGGGCTACAGGCTTGTCACT
GAPDH	TCAACGGATTTGGTCGTATT	CTGTGGTCATGAGTCCTTCC

$$\Delta C_{t(1)} = [C_t(control\ for\ IL6\ or\ TNF{-}\propto) - C_t(GAPDH)]$$

$$\Delta C_{t(2)} = [C_t(control\ for\ IL6\ or\ TNF{-}\propto) - C_t(control\ for\ GADPH)]$$

$$\Delta\Delta C_t = \Delta C_{t(1)} - \Delta C_{t(2)}$$

$$Relative\ expression = 2^{-\Delta\Delta C_t}$$

Where $\Delta C_{t(1)} = deltaC_t$ of unknown sample, $\Delta C_{t(2)} = delta\ C_t$ of control, IL6 and TNF-α = target gene, and GAPDH = housekeeping gene.

Determination of cytokine levels

Serum cytokine levels were determined using a cytokine bead array (CBA) Th1/Th2/Th17 cytokine kit (BD Biosciences, USA). Diluent assay of reconstituting human Th1/Th2/Th17 (2 mL) was used to prepare 10x bulk of cytokines standard (*i.e.*, IFN-γ, TNF-α, IL2, IL4, IL6, IL10, and IL17A). Each cytokine was prepared at the dilution of 1:2, 1:4, 1:8, 1:16, 1:32, 1:64, 1:128, and 1:256. Mixed standards (10 µL) were used for each test. The mixture of beads, standard dilution of each cytokine, and PE Detection Reagent (50 µL) were added to each assay tube and thoroughly mixed. The mixture was incubated (dark, room temperature, 3 h), rinsed with buffer, and centrifuged (200xg, 5 min). The cytokine levels in each sample were measured using flow cytometry and normalized with total blood cell count.

Statistical analysis

Fisher exact and Mann-Whitney U tests with 1,000 bootstrap samples were applied for comparison of proportions and continuous variables, respectively. The correlation between peripheral blood indices and serum cytokine levels was analyzed using Pearson or Spearman for normal and non-normal distributed variables. Receive Operating Characteristics (ROC) analysis was applied to

determine the cut-off values for peripheral blood index and peripheral serum cytokine in iCCA patients compared with healthy subjects. A value of ≥0.71 for the area under the curve (AUC) is considered an acceptable value for evaluating the accuracy of a model. The diagnostic predictors' accuracy was determined by calculating sensitivity and specificity. Data were used for a training set (70%) and validation set (30%), respectively. For missing data, a single imputation was used. All analyses were performed using IBM SPSS statistics (IBM Corp. IBM SPSS statistics for Microsoft Windows, version 29.0, Armonk, New York: IBM). Multi-linear regression analysis was applied using R 4.3.0 program (R core team, 2023). Odds ratios (OR) and AUC are presented as mean ± 95% confident interval (CI). NLR, LMR, PLR, and SIII (x10^9/L) are reported as median (min, max). Sensitivity, specificity, positive predictive value (PPV), negative predictive value (NPV), accuracy index (AI), and Youden index (YI) are reported as mean.

Table S1 Correlations between peripheral blood cytokine levels in healthy subjects.

	**Spearman correlation**	**P-value**
**IFN - TNF**	0.478	0.002
**IFN - IL2**	-0.462	0.003
**IFN - IL4**	-0.037	0.82
**IFN - IL6**	0.232	0.15
**IFN - IL10**	-0.181	0.265
**IFN - IL17A**	0.661	<.001
**TNF - IL2**	-0.057	0.729
**TNF - IL4**	0.454	0.003
**TNF - IL6**	0.16	0.324
**TNF - IL10**	0.308	0.054
**TNF - IL17A**	0.141	0.384
**IL2 - IL4**	0.507	<.001
**IL2 - IL6**	0.236	0.142
**IL2 - IL10**	0.591	<.001
**IL2 - IL17A**	-0.498	0.001
**IL4 - IL6**	0.112	0.492
**IL4 - IL10**	0.805	<.001
**IL4 - IL17A**	-0.426	0.006
**IL6 - IL10**	0.186	0.25
**IL6 - IL17A**	0.167	0.303

**IL10 - IL17A**	-0.517	<.001
**IFN - TNF**	0.478	0.002
**IFN - IL2**	-0.462	0.003
**IFN - IL4**	-0.037	0.82
**IFN - IL6**	0.232	0.15
**IFN - IL10**	-0.181	0.265
**IFN - IL17A**	0.661	<.001
**TNF - IL2**	-0.057	0.729
**TNF - IL4**	0.454	0.003
**TNF - IL6**	0.16	0.324
**TNF - IL10**	0.308	0.054
**TNF - IL17A**	0.141	0.384
**IL2 - IL4**	0.507	<.001
**IL2 - IL6**	0.236	0.142
**IL2 - IL10**	0.591	<.001
**IL2 - IL17A**	-0.498	0.001
**IL4 - IL6**	0.112	0.492
**IL4 - IL10**	0.805	<.001
**IL4 - IL17A**	-0.426	0.006
**IL6 - IL10**	0.186	0.25
**IL6 - IL17A**	0.167	0.303
**IL10 - IL17A**	-0.517	<.001

Table S2 Correlations between peripheral blood cytokine levels in patients with advanced-stage of intrahepatic cholangiocarcinoma.

	**Spearman correlation**	**P-value**
**IFN - TNF**	0.198	0.293
**IFN - IL2**	0.542	0.002
**IFN - IL4**	0.332	0.073
**IFN - IL6**	-0.222	0.239
**IFN - IL10**	0.242	0.197
**IFN - IL17A**	-0.089	0.64
**TNF - IL2**	0.726	<.001

**TNF - IL4**	0.525	0.003
**TNF - IL6**	-0.25	0.183
**TNF - IL10**	-0.293	0.117
**TNF - IL17A**	0.138	0.467
**IL2 - IL4**	0.603	<.001
**IL2 - IL6**	-0.245	0.191
**IL2 - IL10**	-0.077	0.684
**IL2 - IL17A**	-0.182	0.337
**IL4 - IL6**	-0.277	0.139
**IL4 - IL10**	-0.228	0.226
**IL4 - IL17A**	-0.06	0.753
**IL6 - IL10**	0.404	0.027
**IL6 - IL17A**	-0.001	0.995
**IL10 - IL17A**	-0.125	0.51
**IFN - TNF**	0.198	0.293
**IFN - IL2**	0.542	0.002
**IFN - IL4**	0.332	0.073
**IFN - IL6**	-0.222	0.239
**IFN - IL10**	0.242	0.197
**IFN - IL17A**	-0.089	0.64
**TNF - IL2**	0.726	<.001
**TNF - IL4**	0.525	0.003
**TNF - IL6**	-0.25	0.183
**TNF - IL10**	-0.293	0.117
**TNF - IL17A**	0.138	0.467
**IL2 - IL4**	0.603	<.001
**IL2 - IL6**	-0.245	0.191
**IL2 - IL10**	-0.077	0.684
**IL2 - IL17A**	-0.182	0.337
**IL4 - IL6**	-0.277	0.139
**IL4 - IL10**	-0.228	0.226
**IL4 - IL17A**	-0.06	0.753
**IL6 - IL10**	0.404	0.027
**IL6 - IL17A**	-0.001	0.995

**IL10 - IL17A**	-0.125	0.51

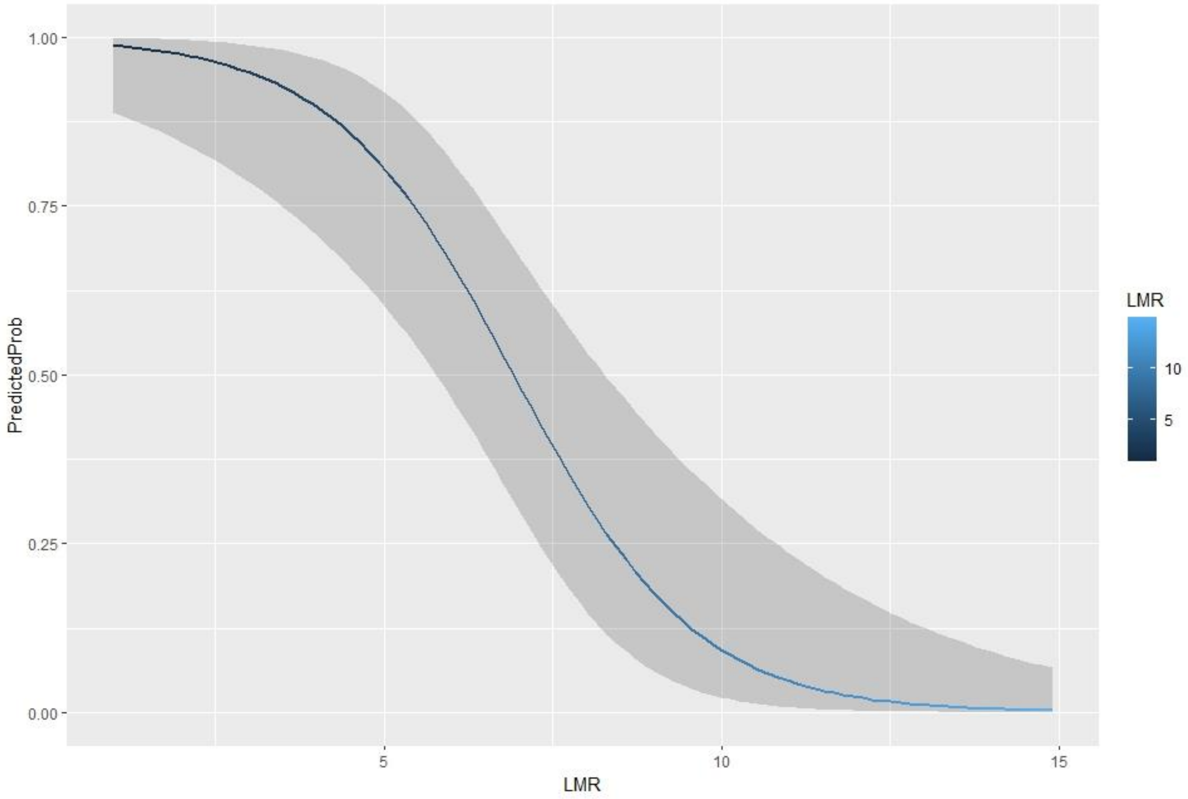

Figure S1. The probability risk of iCCA development and LMR (Data are presented as mean ± 95% confident interval)

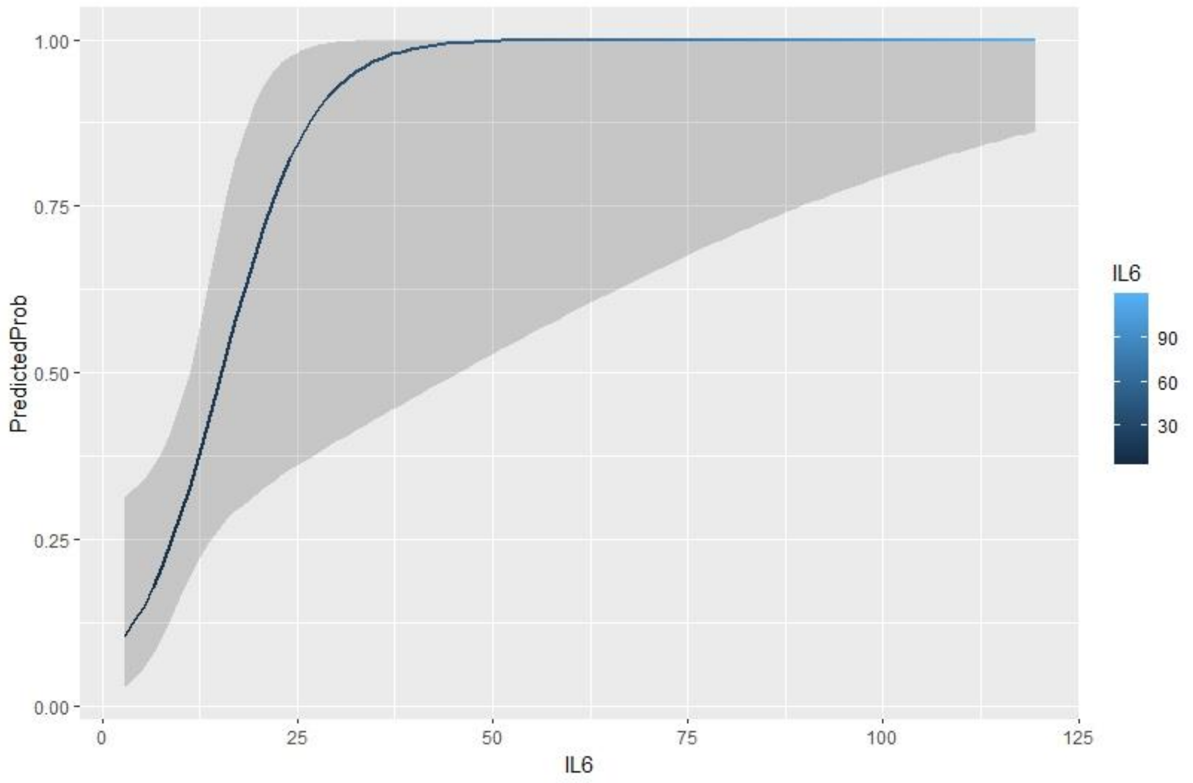

Figure S2. The probability risk of iCCA development and IL6 (Data are presented as mean ± 95% confident interval)

Code for multi-regression analysis and probability risk transformation

Code for LMR

```
myData <- read.csv("C:/Users/itach/Desktop/Phase II PK-PD model/Blood_index_LMR_ONLY.csv")

## view the first few rows of the data

head(myData)

summary(myData)

sapply(mydata,sd)

nrow(myData)

set.seed(1)

sample <- sample(c(TRUE, FALSE), nrow(myData), replace=TRUE, prob=c(0.7,0.3))

train <- myData[sample,]

test <- myData[!sample,]

model_1 <- glm(Group~LMR, data=train, family="binomial")

options(scipen=999)

summary(model_1)
```

```
with(summary(model_1), 1 - deviance/null.deviance)

confint(model_1)

confint.default(model_1)

exp(coef(model_1))

exp(cbind(OR=coef(model_1), confint(model_1)))

newdata1 <- with(myData, data.frame(LMR = median(LMR)))

newdata1$rankP <- predict(model_1, newdata = newdata1, type = "response")

newdata1

newdata2 <- with(myData, data.frame(LMR = rep(seq(from = 1, to = 14.905,length.out = 100),4)))

newdata3 <- cbind(newdata2, predict(model_1, newdata=newdata2, type ="link", se=TRUE))

newdata3 <- within(newdata3, {

  PredictedProb <- plogis(fit)

  LL <- plogis(fit - (1.96 * se.fit))

  UL <- plogis(fit + (1.96 * se.fit))

})

head(newdata3)

library(plyr)

library(ggplot2)

ggplot(data=newdata3, aes(x=LMR, y=PredictedProb)) + geom_ribbon(aes(ymin=LL, ymax=UL),
alpha=0.2) + geom_line(mapping=aes(colour=LMR), size=1)

# View(newdata3)

#write.table(newdata3, file="ExportedFileName.csv", sep=",")
```

Code for IL6

```
myData1 <- read.csv("C:/Users/itach/Desktop/Phase II PK-PD model/Interleukin.csv")

## view the first few rows of the data

head(myData1)

summary(myData1)

sapply(myData1,sd)

nrow(myData1)

set.seed(1)

sample <- sample(c(TRUE, FALSE), nrow(myData1), replace=TRUE, prob=c(0.7,0.3))
```

```
train <- myData1[sample,]

test <- myData1[!sample,]

model2 <- glm(GROUP~IL6, data=train, family="binomial")


summary(model2)

with(summary(model2), 1 - deviance/null.deviance)

confint(model2)

confint.default(model2)

exp(coef(model2))

exp(cbind(OR=coef(model2), confint(model2)))

newdata1 <- with(myData1, data.frame(IL6=median(IL6)))

newdata1$rankP <- predict(model2, newdata = newdata1, type = "response")

newdata1

newdata2 <- with(myData, data.frame(IL6 = rep(seq(from = 3, to = 119.51,length.out = 100),4)))

newdata3 <- cbind(newdata2, predict(model2, newdata=newdata2, type ="link", se=TRUE))

newdata3 <- within(newdata3, {

  PredictedProb <- plogis(fit)

  LL <- plogis(fit - (1.96 * se.fit))

  UL <- plogis(fit + (1.96 * se.fit))

})

head(newdata3)

library(plyr)

library(ggplot2)

ggplot(data=newdata3, aes(x=IL6, y=PredictedProb)) + geom_ribbon(aes(ymin=LL, ymax=UL),
alpha=0.2) + geom_line(mapping=aes(colour=IL6), size=1)

# View(newdata3)

#write.table(newdata3, file="ExportedFileName.csv", sep=",")
```

Please cite this article for using the R code.
